# A Nomogram for Predicting Pathological Complete Response to Neoadjuvant Chemoradiotherapy Using Semiquantitative Parameters Derived From Sequential PET/CT in Locally Advanced Rectal Cancer

**DOI:** 10.3389/fonc.2021.742728

**Published:** 2021-10-05

**Authors:** Dae Hee Pyo, Joon Young Choi, Woo Yong Lee, Seong Hyeon Yun, Hee Cheol Kim, Jung Wook Huh, Yoon Ah Park, Jung Kyong Shin, Yong Beom Cho

**Affiliations:** ^1^ Department of Surgery, Samsung Medical Center, Sungkyunkwan University School of Medicine, Seoul, South Korea; ^2^ Department of Nuclear Medicine, Samsung Medical Center, Sungkyunkwan University School of Medicine, Seoul, South Korea; ^3^ Department of Health Sciences and Technology, Samsung Advanced Institute for Health Sciences & Technology (SAIHST), Sungkyunkwan University, Seoul, South Korea; ^4^ Department of Biopharmaceutical Convergence, Sungkyunkwan University, Seoul, South Korea

**Keywords:** locally advanced rectal cancer (LARC), neoadjuvant chemoradiotherapy, pathological complete response (pCR), PET/CT, nomogram

## Abstract

We evaluated the predictive value of semiquantitative volumetric parameters derived from sequential PET/CT and developed a nomogram to predict pathological complete response (pCR) in patients with rectal cancer treated by neoadjuvant chemoradiotherapy (nCRT). From April 2008 to December 2013, among the patients who underwent nCRT, those who were taken sequential PET/CT before and after nCRT were included. MRI-based staging and semiquantitative parameters of PET/CT including standardized uptake value (SUV), metabolic tumor volume (MTV), and total lesion glycolysis (TLG) were evaluated before and after nCRT. Multivariable analysis was performed to select significant predictors to construct a nomogram. Sensitivity, specificity, accuracy, and area under the receiver operating characteristics curve (AUC) of the model were evaluated to determine its performance. Among 137 eligible patients, 17 (12.4%) had pCR. All post-PET/CT parameters showed significant differences between pCR and non-pCR groups. Patients were randomly assigned to a training group (91 patients) and a validation group (46 patients). In multivariable analysis with the training group, post-CEA, post-MRI T staging, post-SUV_max_, and post-MTV were significantly associated with pCR. There was no significant pre-nCRT variable for predicting pCR. Using significant predictors, a nomogram was developed. Sensitivity, specificity, accuracy, and AUC of the nomogram were 0.882, 0.808, 0.848, and 0.884 with the training group and 0.857, 0.781, 0.783, and 0.828 with the validation group, respectively. This model showed the better performance than other predictive models that did not contain PET/CT parameters. A nomogram containing semiquantitative post-PET/CT could effectively select candidates for organ-sparing strategies.

## Introduction

Colorectal cancer is one of the most commonly diagnosed malignancies. It is the major cause of cancer-related deaths in the world according to reports of the World Health Organization (1). In 2020, 732,210 new cases of rectal cancer were diagnosed with 339,022 deaths due to rectal cancer. The current standard treatment for non-metastatic locally advanced rectal cancer (LARC) is a preoperative or neoadjuvant long-course concurrent chemoradiotherapy (nCRT) followed by radical surgery at intervals of 8–12 weeks (2). After completion of nCRT, approximately 15–20% patients achieve pathological complete response (pCR) defined as an absence of any residual cancer cells (ypTxN0M0) in the surgical specimen (3, 4). Because radical surgery for rectal cancer causes significant morbidity and deteriorates patients’ quality of life, causing fecal, urinary, or sexual dysfunction and permanent stoma in some cases, organ-sparing strategies such as “wait-and-see” (5–7) and transanal local excision (8–10), have been recently proposed. One of the most important prerequisites to select appropriate candidates for these conservative strategies is the construction of a reliable prediction model for pCR without pathological information of surgical specimens. Although many efforts have been made to identify robust clinical predictors for pCR, any single modality has not been validated to present a sufficient predictive power. Although serum level of carcinoembryonic antigen (CEA) could be easily and rapidly evaluated, its false-positive rates cause concerns (11). Magnetic resonance imaging (MRI) has advantages on excellent spatial resolution enabling anatomical diagnosis for depth of tumor invasion and identification of lymph nodes (12). However, without diffusion and intravenous contrast MRI has a limited role in evaluating the viability of tumor.

PET/CT is a well-established imaging modality for cancer evaluation. It is advantageous in presenting the physiological process of a tumor, thereby distinguishing the remained viable tumor tissue from the fibrosis induced by radiation. Recent studies have revealed that several semiquantitative metabolic and volumetric parameters derived from PET/CT, including metabolic tumor volume (MTV), total lesion glycolysis (TLG), and standardized uptake value (SUV), are significantly associated with therapeutic responses in several types of cancer (13–16).

In this study, we evaluated the predictive efficacy of semiquantitative metabolic and volumetric parameters derived from sequential PET/CT taken before and after nCRT in patients with LARC. In addition, we developed and validated a pCR-predicting nomogram incorporating PET/CT parameters with other clinical features including CEA and MRI findings.

## Materials and Methods

### Patient Selection

Among non-metastatic primary rectal cancer patients with clinical T3/T4 stage, or lymph node involvement treated with nCRT followed by curative resection at Samsung Medical Center from April 2008 to December 2013, those who underwent sequential PET/CTs taken before and after nCRT were included in this study. All patients were staged with standard examinations at the initial workup, including digital rectal examination, endoscopic ultrasound, rigid proctoscopy, abdominopelvic computed tomography (CT), pelvic MRI, serum level of CEA, and PET/CT. After completion of nCRT, blood test for CEA, MRI, and PET/CT were performed. Informed consent was obtained from all participants to preserve their clinical data in a form of a database to use in future research regarding colorectal cancer. Data were extracted from the Clinical Data Warehouse Darwin-C of Samsung Medical Center for this study. This retrospective study design was approved by the Institutional Review Board (IRB) of Samsung Medical Center (Number: 2019-12-056).

### Neoadjuvant Chemoradiotherapy and Surgery

The use of nCRT was decided by a multidisciplinary team consisting of colorectal surgeons, medical oncologists, and radiation oncologists. Radiation was administered to the whole pelvic field at a total dose of 50.4 Gy in 25 fractions. Chemotherapy was administered concurrently with radiation based on 5-fluorouracil (5-FU) or capecitabine. 5-FU (425 mg/m^2^/day) and leucovorin (20 mg/m^2^/day) were administered intravenously for 5 days during the first and fifth weeks of radiotherapy. Oral capecitabine (825 mg/m^2^/day) was administered twice daily during the period of radiotherapy. All patients underwent curative resection with 8 weeks of intervals from the completion of nCRT. Surgery was performed by experienced colorectal surgeons following principles of total mesorectal excision.

### MRI Staging and Pathological Staging

All MRI reports were retrospectively reviewed. Tumors with definite invasion to mesorectal fascia were defined as T4 stage. Tumors with invasion into perirectal fat tissues without reaching the mesorectal fascia were defined as T3 stage. Tumors without evidence of invasion to perirectal fat tissue and confined in muscle layer or within the mucosa were defined as T1–T2. Tumors with one or more probable or definite metastatic lymph node enlargement were defined as N+. Pathological CR was defined as ypTxN0M0.

### ^18^F-FDG PET/CT Imaging and Interpretation

Baseline 18F-FDG PET/CT was performed at 7–10 days before the induction of nCRT. Follow-up PET/CT was performed at 4–5 weeks after the completion of nCRT. Patients fasted for at least 6 h before the PET/CT study. Blood glucose levels were measured. They were required to be <200 mg/dl. Whole-body PET and unenhanced CT images were acquired using a PET/CT scanner (Discovery STE, GE Healthcare, Milwaukee, WI, USA). Whole-body CT was performed using a 16-slice helical CT with 30–170 mAs adjusted to the patient’s body weight at 140 kVp and 3.75-mm section width. After the CT scan, an emission scan was performed from the thigh to the head for 2.5 min per frame in three-dimensional mode, at 60 min after the intravenous injection of 18F-FDG (5.5 MBq/kg). PET images were reconstructed using CT for attenuation correction using ordered subsets expectation–maximization algorithm (20 subsets, two iterations) with a voxel size of 3.9 × 3.9 × 3.3 mm. The SUV was normalized to the patient’s body weight. Volume-based assessments of 18F-FDG PET/CT were performed using a volume viewer software on a GE Advantage Workstation version 4.4. We placed a volume of interest over the primary tumor using a threshold SUV of 2.5 for tumor segmentation because this cutoff value is generally considered to be indicative of malignant tissue regardless of the scanner (15). The software then measured SUVmax, mean SUV (SUV_mean_), a standard deviation of SUV (SUV_sd_), and MTV. TLG was calculated by multiplying SUV_mean_ by MTV. Δ value was defined as the difference between pre-PET/CT parameters and post-PET/CT parameters divided by pre-PET/CT parameters.

### Statistical Analysis

Statistical differences between groups were calculated using Student’s t-tests for continuous variables and χ^2^ test or Fisher’s exact test for categorical variables. Patients were divided to training and validation groups by random sampling with a ratio of 2:1. Univariable logistic regression analysis for the training group was performed with cell differentiation, and pre- and postvalue of CEA, MRI T stage, MRI N stage, SUV_max_, SUV_mean_, SUV_sd_, MTV, and TLG. Multivariable regression analysis for the training group was performed using variables showing significant associations (*p* < 0.05) with pCR in univariable regression analysis.

Patients were randomly assigned to a training group or a validation group with a ratio of 2:1. Predictive models were constructed using a training group and evaluated the efficacy in a validation group. A nomogram was established based on results of multivariable regression analysis. Other models that excluded PET/CT parameters in explanatory variables were also fitted and compared with the nomogram. The model containing CEA only, CEA with MRI staging, and CEA with MRI staging and PET/CT parameters as explanatory variables were named as “CEA” model, “CEA + MRI” model, and “CEA + MRI + PET/CT” model, respectively. Performances of these models were evaluated in terms of sensitivity, specificity, accuracy, and area under the receiver operating characteristic curve (AUC). Survival analyses were performed using the Kaplan–Meier method. Survival differences between groups were evaluated using the log-rank test. All statistical analyses were performed using R version 3.5.0. software (http://www.r-project.org, R Foundation for Statistical Computing, Vienna, Austria). All *p* < 0.05 were considered statistically significant.

## Results

Among the 318 patients with rectal adenocarcinoma who underwent curative resection, 145 (45.6%) performed sequential ^18^F-FDG PET/CT before and after nCRT. After excluding 3 (2.1%) patients who underwent emergent surgery due to obstruction, 3 (2.1%) patients who had multiple primary colorectal cancers, and 2 (1.4%) patients who were diagnosed as metastatic diseases at the post-PET/CT, a total of 137 patients were finally recruited (**Table 1**). The number of patients who achieved pCR was 17 (12.4%). The median pre-CEA was 1.5 ng/ml in the pCR group and 2.9 ng/ml in the non-pCR group (*p* = 0.005). The pCR group also had significantly lower post-CEA (1.0 vs. 1.6 ng/ml, *p* = 0.012). The number of patients with post-MRI Tx was 8 (47.1%) in the pCR group and 17 (14.2%) in the non-pCR group (*p* = 0.001). Proportions of patients with post-MRI N(−) stage were not significantly different between the two groups (17.6% vs. 5.0%, *p* = 0.148). Moreover, pre-PET/CT parameters showed no significant differences between the two groups. However, post-SUV_max_, SUV_mean_, SUV_sd_, MTV, and TLG were significantly lower in the pCR group than in the non-pCR group.

**Table 1:** Comparisons of clinicopathological characteristics of patients who achieved pathological complete response and those who did not.

	pCR (n = 17)	Non-pCR (n = 120)	*p*-value
Age, n (%)			0.368
≥65	2 (11.8)	30 (25.0)	
<65	15 (88.2)	90 (75.0)	
Sex, n (%)			0.982
Male	11 (64.7)	82 (68.3)	
Female	6 (35.3)	38 (31.7)	
BMI, mean (SD), kg/m^2^	25.1 (3.4)	24.2 (3.0)	
Pre-CEA, median (IQR), ng/ml	1.5 (1.1–2.6)	2.9 (1.7–4.1)	0.005
Post-CEA, median (IQR), ng/m	1.0 (0.6–1.5)	1.6 (1.0–2.3)	0.012
Cell differentiation, n (%)			1.000
WD/MD	15 (88.2)	104 (86.7)	
PD/Mucinous	2 (11.8)	16 (13.3)	
Pre-MRI T stage, n (%)			0.129
T1 or T2	6 (35.3)	19 (15.8)	
cT3	8 (47.1)	64 (53.3)	
cT4	3 (17.6)	37 (30.8)	
Pre-MRI N stage, n (%)			0.821
N−	1 (5.9)	2 (1.7)	
N+	16 (94.1)	118 (98.3)	
Post-MRI T stage, n (%)			0.001
Tx	8 (47.1)	17 (14.2)	
T1 or T2	6 (35.3)	21 (17.5)	
T3	2 (11.8)	65 (54.2)	
T4	1 (5.9)	17 (14.2)	
Post-MRI N stage, n (%)			0.148
N−	3 (17.6)	6 (5.0)	
N+	14 (82.4)	114 (95.0)	
Pathologic T stage, n (%)			<0.001
ypTx	17 (100)	2 (1.7)	
ypT1	0	4 (3.3)	
ypT2	0	50 (41.7)	
ypT3	0	63 (52.5)	
ypT4	0	1 (0.8)	
Pathologic N stage, n (%)			0.01
ypN0	17 (100)	76 (63.3)	
ypN1	0	34 (28.3)	
ypN2	0	10 (8.3)	
Pre-SUV_max_, median (IQR)	13.3 (10.7–16.6)	14.4 (9.7–17.9)	0.739
Pre-SUV_mean_, median (IQR)	6.2 (4.9–7.8)	8.0 (4.9–9.2)	0.195
Pre-SUV_sd_, median (IQR)	2.8 (1.8–3.5)	2.3 (1.8–2.9)	0.601
Pre-MTV, median (IQR)	18.0 (6.1–29.5)	19.8 (14.0–32.6)	0.245
Pre-TLG, median (IQR)	77.0 (55.9–236.2)	122.3 (75.7–216.1)	0.249
Post-SUV_max_, median (IQR)	3.1 (2.2–4.6)	6.8 (4.0–9.8)	0.005
Post-SUV_mean_, median (IQR)	2.8 (2.6–3.2)	3.2 (2.7–3.9)	0.035
Post-SUV_sd_, median (IQR)	0.4 (0.4–0.6)	0.7 (0.5–1.0)	<0.001
Post-MTV, median (IQR)	2.4 (1.3–4.8)	6.1 (3.8–12.7)	0.020
Post-TLG, median (IQR)	0.5 (3.8–13.0)	12.8 (6.0–33.1)	0.019
ΔSUV_max_, median (IQR)	72.1 (57.5–76.5)	60.0 (48.2–69.8)	0.015
ΔSUV_mean_, median (IQR)	60.6 (43.9–72.2)	44.7 (34.1–58.3)	0.022
ΔSUV_sd_, median (IQR)	81.2 (66.3–87.5)	66.7 (54.9–78.5)	0.005
ΔMTV, median (IQR)	82.9 (50.3–92.1)	79.5 (57.9–90.8)	0.659
ΔTLG, median (IQR)	93.8 (79.1–96.8)	87.7 (75.7–95.2)	0.221

pCR, pathological complete response; BMI, body mass index; SD, standard deviation; CEA, carcinoembryonic antigen; IQR, interquartile range; WD, well differentiation; MD, moderately differentiation; PD, poorly differentiation; MRI, magnetic resonance imaging; SUV, standardized uptake value; MTV, metabolic tumor volume; TLG, total lesion glycolysis.

**Figure 1:**
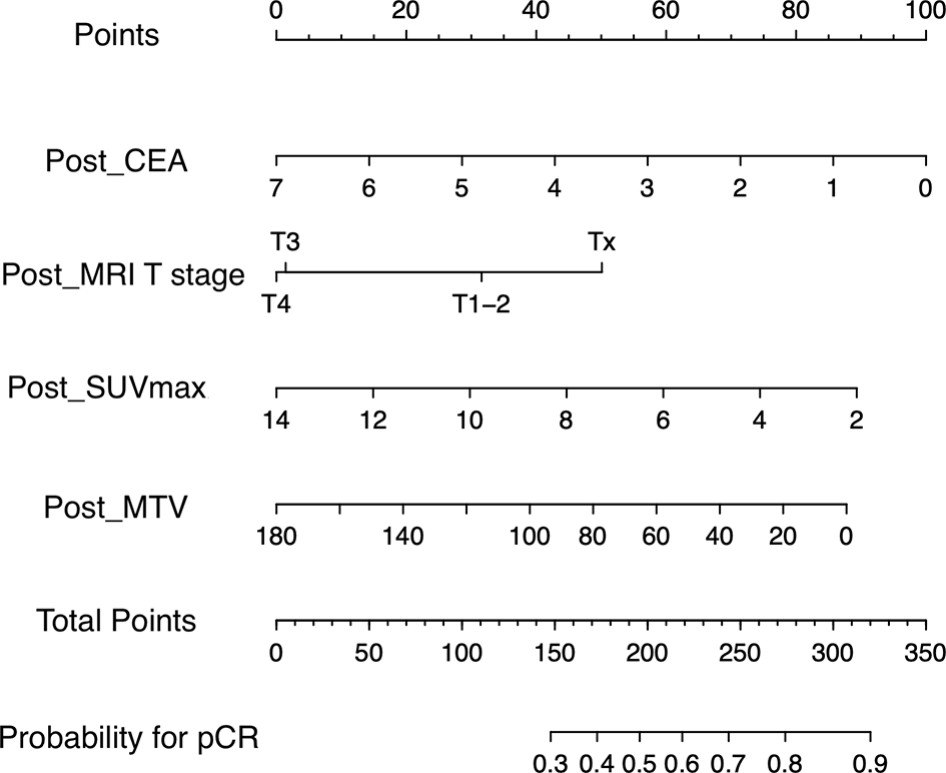
Nomogram for predicting pathologic complete response. A point for each predictor can be read out along the top scale bar, ‘Points’. The sum of points or total points is converted to the ‘probability for pCR. pCR’ (CEA, carcinoembryonic antigen; MRI, magnetic resonance imaging; SUVmax, maximum value of standardized uptake value; MTV, metabolic tumor volume; pCR, pathologic complete response).

**Figure 2:**
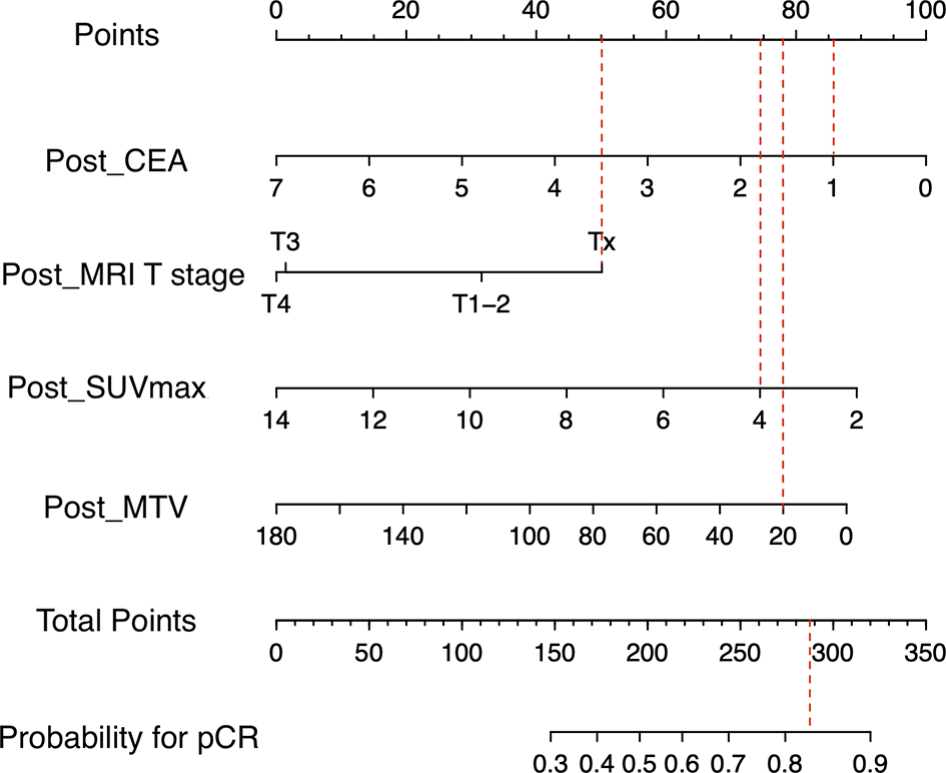
Application of nomogram for a virtual patient.

In comparison between training group containing 91 (66.4%) patients and validation group containing 46 (33.6%) patients, age, sex, body mass index, pre- and post-CEA, cell differentiation, pre- and post- MRI T and N staging, and pre- and post- PET/CT parameters showed no significant. The rate of pCR was 11.0% (10/91) in the training group and 15.2% (7/46) in the validation group. Univariable regression analysis of the training group revealed that pre-CEA, post-CEA, post-MRI T staging, post-SUVmax, post-SUVmean, post-MTV, post-TLG, and ΔSUV_max_ were significantly correlated with pCR. In multivariable regression analysis using these variables, post-CEA, post-MRI T staging, post-SUV_max_, and post-MTV were independent predictors for pCR (**Table 2**). A nomogram incorporating these independent predictors was developed (**Figure 1**). Each value or category within these factors was assigned a score on the point scale bar. After obtaining the total score, a vertical line was drawn downwards from the total point scale bar to produce probability for pCR. For example, suppose a virtual patient whose post-CEA is 1 ng/ml, post-MRI T stage is Tx, post-SUV_max_ is 4, and post-MTV is 20 (**Figure 2**). The points for each item are 86, 50, 74, and 78, respectively, and the total point is 288. Finally, the probability for pCR corresponding to the total point of 288 is 0.82.

**Table 2:** Multivariable regression models for pathologic complete response in the training group.

	Odds ratio	95% confidence interval	*p*-value
Post-CEA	2.503	1.107–6.918	0.048
Post-MRI T stage			
T1–2 *vs*. Tx	0.960	0.240–3.823	0.954
T3 *vs*. Tx	5.312	1.878–64.93	0.011
T4 *vs*. Tx	8.893	0.730–110.0	0.152
Post-SUV_max_	1.547	1.068–2.493	0.041
Post-MTV	1.187	1.113–1.486	0.039

CEA, carcinoembryonic antigen; MRI, magnetic resonance imaging; SUV, standardized uptake value; MTV, metabolic tumor volume.

**Figure 3:**
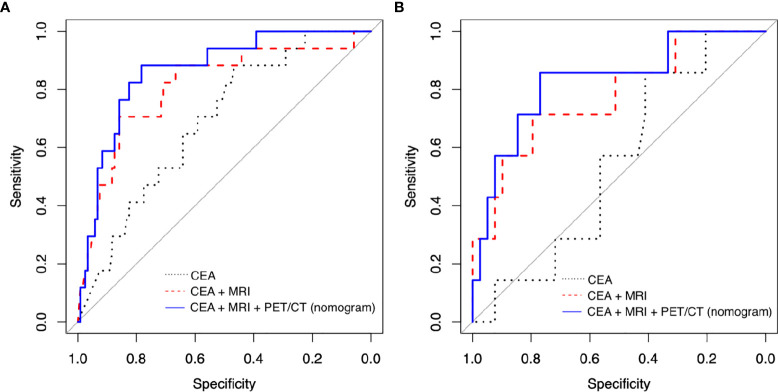
Receiver operating characteristic (ROC) curve analysis to evaluate the predictive power of models in **(A)** training group and **(B)** validation group. (CEA, carcinoembryonic antigen; MRI, magnetic resonance imaging; PET/CT, positron emission tomography/computed tomography).

Sensitivity, specificity, accuracy, and AUC of nomogram were 0.882, 0.808, and 0.884, respectively (**Table 3** and **Figure 3A**). To validate the nomogram, it was adopted to patients in the validation group to evaluate the performance (**Table 4** and **Figure 3B**). Sensitivity, specificity, accuracy, and AUC of the nomogram were 0.857, 0.781, 0.783, and 0.828, respectively.

**Table 3:** Performances of models for the training group.

	Sensitivity	Specificity	Accuracy	AUC
CEA	0.706	0.467	0.518	0.689
CEA + MRI	0.882	0.858	0.839	0.831
CEA + MRI + PET/CT (nomogram)	0.882	0.808	0.848	0.884

CEA, carcinoembryonic antigen; MRI, magnetic resonance imaging; PET/CT, positron emission tomography/computed tomography.

**Table 4:** Performances of models for the validation group.

	Sensitivity	Specificity	Accuracy	AUC
CEA	0.857	0.410	0.501	0.544
CEA + MRI	0.714	0.795	0.783	0.777
CEA + MRI + PET/CT (nomogram)	0.857	0.781	0.783	0.828

CEA, carcinoembryonic antigen; MRI, magnetic resonance imaging; PET/CT, positron emission tomography/computed tomography.

Without PET/CT parameters, we also construed other prediction models including “CEA” model and “CEA + MRI” model using the training group. “CEA” model contained post-CEA only, and “CEA + MRI” model had post-CEA with post-MRI T staging as explanatory variables. AUC was 0.689 for the “CEA” model and 0.831 for the “CEA + MRI” model, lower than that of the nomogram at 0.884 (**Table 3** and **Figure 3A**). With the validation group, the AUC was 0.544 for the “CEA” model and 0.777 for the “CEA + MRI” model, also lower than that of the nomogram at 0.828 (**Table 4** and **Figure 3B**).

**Figure 4:**
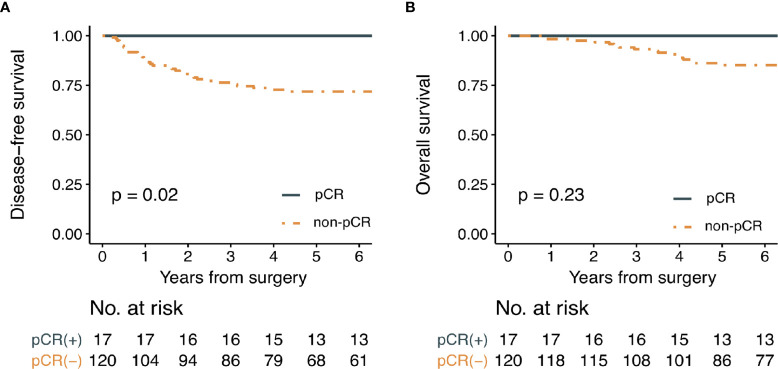
Kaplan-Meier survival curve analysis for **(A)** disease-free survival and **(B)** overall survival in patients with or without pathologic complete response. (pCR; pathologic complete response).

The median follow-up period was 87 months. Oncological outcomes were compared between pCR and non-pCR groups (**Figure 4**). Three-year disease-free survival rate was 100% for the pCR group and 76.3% for the non-pCR group (*p* = 0.02). Three-year overall survival was 100% for the pCR group and 93.2% for the non-pCR group (*p* = 0.23).

## Discussion

The clinical evidence for excellent prognosis of patients with pCR has been well established (17). Our data also revealed that 5-year disease-free survival and overall survival of patients with pCR were 100%. Therefore, we could infer that oncological outcomes of these patients may not be compromised by the application of organ-sparing strategies. To select these patients accurately, we considered several clinical variables. Most of all, this study evaluated predictive values of semiquantitative volumetric and metabolic parameters derived from pre- and post-PET/CT for pCR in patients with LARC who underwent nCRT. Our findings demonstrated that not pre-PET/CT, but post-PET/CT parameters were significantly correlated with pCR. Our results also revealed that post-SUV_max_ and post-MTV and CEA and post-MRI T staging were independent predictors in multivariable regression analysis. A nomogram incorporating post-PET/CT parameters with post-CEA and post-MRI T staging features was successfully developed and validated, with predictive performances of AUC 0.884 and 0.828 for the training group and the validation group, respectively. Because performances of the nomogram were better than other models that did not contain PET/CT parameters, the addition of PET/CT variables, especially post-SUV_max_ and MTV, could improve a model’s predictive power for pCR.

Interestingly, our results demonstrated that pre-nCRT variables were not correlated with pCR in multivariable regression. Ryan et al. have performed a systematic review for predicting pCR using pre-nCRT variables in LARC (18). They selected 85 articles addressing the prediction of pCR with clinical, radiological, and molecular characteristics. Although some studies in their review suggested that pre-CEA, pre-MRI parameter, specific mutation profiles, and/or protein expression profiles of tumors were associated with pCR, no robust solitary pre-nCRT marker was identified. Moreover, no studies have confirmed the significant predictability of pre-PET/CT parameters for pCR in the review, corresponding to results of the present study.

Because radiation-related tumor shrinkage effect is time-dependent phenomenon, the optimal timing of restaging and surgery after nCRT completion has long been a critical issue. Although a minimum of 6–8 weeks interval to surgery is commonly recommended to maximize a tumor downsizing and pCR rates, a consensus or clinical guidelines regarding the best timing for assessment of tumor response to nCRT is still lacking. Perez et al. conducted a clinical trial to estimate the metabolic activity at 6 and 12 weeks after nCRT by PET/CT (19). The patients were treated with long-course nCRT and underwent three PET/CT at baseline, 6 weeks, and 12 weeks from nCRT completion. In the results of the study, SUV_max_ decreased until 6 weeks for both good responders and bad responders, remained identical or further decreased from 6 to 12-week PET/CT imaging for good responders, and showed a rise from 6 to 12-week PET/CT imaging for bad responders. This study also showed that a decrease between early (1 h) and late (3 h) SUV_max_ at 6-week PET/CT imaging could predict good responders with an accuracy of 67%. Gasinska et al. also showed that repopulation of tumor cells occurred 4 weeks after nCRT completion (20). In this study, post-PET/CT was conducted 4–5 weeks after nCRT completion based on the results of the previously said studies. However, to establish robust evidence for an optimal timing for reassessment by PET/CT after long-course nCRT completion, a well-designed randomized controlled trials should be conducted.

Although follow-up or restaging imaging with MRI has been routinely recommended in clinical guidelines, the clinical benefit and usefulness of restaging PET/CT have yet to be established (12, 21, 22). Recently, some studies have shown that the predictive power of post-nCRT variables may be better than those of pre-nCRT variables, meaning that post-nCRT clinical or imaging features could provide more valuable information regarding the response to nCRT (23–27). Moreover, restaging with PET/CT could even detect new metastatic lesions after long-course nCRT in some patients with non-pCR (25).

However, as mentioned above, no modality including MRI or PET/CT was confirmed as a single significant predictor for pCR. Therefore, researchers tried to integrate several markers to improve the performance of predicting models. Ren et al. have constructed a nomogram for predicting pCR in patients treated by neoadjuvant mFOLFOX6 with radiotherapy, known as total neoadjuvant therapy (TNT) (28). These patients were participants in the FOWARC trial (29). Their nomogram contained variables of tumor differentiation, mesorectal fascia status evaluated by pre-MRI, regimen of nCRT, and tumor size. However, they did not consider PET/CT parameters. Although their nomogram showed good statistical performance for predicting the probability of pCR with C-index of 79.34%, it might be due to a relatively high pCR rate (17.9%) caused by more aggressive neoadjuvant therapy regimen compared to standard nCRT. Considering that high pCR rate itself could improve the accuracy of predicting models in statistics, our nomogram showing an accuracy of 78.3% in the patient cohort with pCR rate of 12.4% might have potential to show better performance in the patient cohort treated by TNT known to induce a higher pCR rate.

It has been known that MRI and PET/CT have comparable diagnostic performance for the prediction of pCR (30). Joye et al. have conducted a systematic review for studies on the role of diffusion-weighted MRI and PET/CT in the prediction of pCR and concluded that diffusion-weighted MRI or PET/CT alone is not accurate in prediction of pCR, although it has strength in the identification of non-pCR (31). In their study, integration of MRI and PET/CT was not considered. Because both modalities showed complimentary results in many studies, 18-F FDG PET/MRI was suggested as a solution to increase the sensitivity by adding MRI parameters to PET parameter, and the initial experience was reported recently (32). However, this technique has some disadvantages compared to other hybrid imaging techniques including lack of protocol and standardization, limited flexibility of combined PET/MRI systems, and requirement of high cost. In addition, several technical challenges such as the addition of PET components to the system in the presence of strong magnetic field from MR have remained to be widely used in clinical practices (33).

As PET/MR technically integrates PET and MRI, our nomogram statistically integrates their outputs. Because post-MRI could precisely determine the tumor’s depth of invasion, post-MRI T staging was a significant predictor for pCR in our study. However, the accuracy of post-MRI N staging was limited because MRI could only assess the size and shape of a lymph node instead of its physiologic activity. This limitation of MRI was supplemented by semiquantitative parameters of post-PET/CT. SUV_max_, the maximum voxel value of SUV in the target lesion, is the most valuable and common parameter of PET/CT. However, it does not reproduce the whole metabolic tumor burden. In addition, it is vulnerable to various noises generated by several factors, including patient characteristics (34). MTV is a measurement of functional tumor volume with high metabolic activity. TLG is a product of MTV and mean SUV. These semiquantitative volumetric parameters could represent metabolic activity of the tumor better than SUV_max_ (13–16). In recent years, several studies have analyzed predictive values of MTV and TLG for pCR in LARC (35–37). However, no parameter alone was sufficiently effective to play a secure role in selecting patients with pCR. For the above-mentioned reasons, we incorporated all parameters derived from PET/CT with MRI features into the nomogram.

This study had some limitations. First, because this study was conducted retrospectively and the cohort did not represent all consecutive patients with LARC treated in this center, the inclusion of patients might have been affected by selection bias. Second, results of this single-center analysis based on a small number of patients lacked generalizability. Especially, an external validation using a test group or patients outside this center was not performed. Third, calculating parameters of PET/CT was laborious to be easily applied to real-world practice. Moreover, as it was performed by expert nuclear medicine physicians, it may raise concerns regarding interobserver variability issues. Further well-designed multicenter prospective studies are warranted to confirm the predictive role of this nomogram. Fourth, because the PET/CT has fundamentally limited performance on spatial resolution and the resulting partial volume effect, PET/CT parameters of the small lesions may be underestimated, and this false negativity may exaggerate the probability for pCR in a nomogram. Therefore, if the post-SUV_max_ or post-MTV of the lesion was too low or not detected while post-MRI T stage was obviously greater than T1–T2, the results of nomogram should be cautiously interpreted.

In conclusion, post-PET/CT parameters including post-SUV_max_ and post-MTV have significant predictive values for pCR. A nomogram incorporating semiquantitative post-PET/CT parameters with post-MRI features could effectively select candidates for organ-sparing strategies.

## Data Availability Statement

The raw data supporting the conclusions of this article will be made available by the authors, without undue reservation.

## Ethics Statement

The studies involving human participants were reviewed and approved by Samsung Medical Center. The patients/participants provided their written informed consent to participate in this study.

## Author Contributions

DHP performed the statistical analysis and data interpretation and wrote a draft. JYC calculated the parameters of PET/CT. YBC designed the core conception and guided the whole process. YBC, WYL, SHY, HCK, JWH, YAP, and JKS constructed and collected the clinical database. YBC did a critical revision for intellectual content. All authors contributed to the article and approved the submitted version.

## Funding

This research was supported by a grant of the Korea Health Technology R&D project through the Korea Health Industry Development Institute (KHIDI), funded by the Ministry of Health & Welfare, Republic of Korea (grant number: HR20C0025). This work was supported by the BK21 FOUR Project.

## Conflict of Interest

The authors declare that the research was conducted in the absence of any commercial or financial relationships that could be construed as a potential conflict of interest.

## Publisher’s Note

All claims expressed in this article are solely those of the authors and do not necessarily represent those of their affiliated organizations, or those of the publisher, the editors and the reviewers. Any product that may be evaluated in this article, or claim that may be made by its manufacturer, is not guaranteed or endorsed by the publisher.
